# Characterization of Enteroviruses from Non-Human Primates in Cameroon Revealed Virus Types Widespread in Humans along with Candidate New Types and Species

**DOI:** 10.1371/journal.pntd.0003052

**Published:** 2014-07-31

**Authors:** Serge Alain Sadeuh-Mba, Maël Bessaud, Marie-Line Joffret, Marie-Claire Endegue Zanga, Jean Balanant, Eitel Mpoudi Ngole, Richard Njouom, Jean-Marc Reynes, Francis Delpeyroux, Dominique Rousset

**Affiliations:** 1 Service de Virologie, Centre Pasteur du Cameroun, Yaounde, Cameroon; 2 Institut Pasteur, Unité de Biologie des Virus Entériques, Paris, France; 3 INSERM, U994, Paris, France; 4 Projet Prévention du Sida au Cameroun (PRESICA), Yaounde, Cameroon; Beijing Institute of Microbiology and Epidemiology, China

## Abstract

Enteroviruses (EVs) infecting African Non-Human Primates (NHP) are still poorly documented. This study was designed to characterize the genetic diversity of EVs among captive and wild NHP in Cameroon and to compare this diversity with that found in humans. Stool specimens were collected in April 2008 in NHP housed in sanctuaries in Yaounde and neighborhoods. Moreover, stool specimens collected from wild NHP from June 2006 to October 2008 in the southern rain forest of Cameroon were considered. RNAs purified directly from stool samples were screened for EVs using a sensitive RT-nested PCR targeting the VP1 capsid coding gene whose nucleotide sequence was used for molecular typing. Captive chimpanzees (*Pan troglodytes*) and gorillas (*Gorilla gorilla*) were primarily infected by EV types already reported in humans in Cameroon and elsewhere: Coxsackievirus A13 and A24, Echovirus 15 and 29, and EV-B82. Moreover EV-A119, a novel virus type recently described in humans in central and west Africa, was also found in a captive Chimpanzee. EV-A76, which is a widespread virus in humans, was identified in wild chimpanzees, thus suggesting its adaptation and parallel circulation in human and NHP populations in Cameroon. Interestingly, some EVs harbored by wild NHP were genetically distinct from all existing types and were thus assigned as new types. One chimpanzee-derived virus was tentatively assigned as EV-J121 in the EV-J species. In addition, two EVs from wild monkeys provisionally registered as EV-122 and EV-123 were found to belong to a candidate new species. Overall, this study indicates that the genetic diversity of EVs among NHP is more important than previously known and could be the source of future new emerging human viral diseases.

## Author Summary

Enteroviruses (EVs) are among the most common viruses infecting humans. The circulation and diversity of EVs infecting humans have been extensively studied in most parts of the world but those infecting Non-Human Primates (NHP) are still poorly documented. As most African countries, Cameroon houses a wide range of Old World primates' species including apes (chimpanzees and gorillas) and at least 28 species of Old World monkeys. In this study, we investigated the diversity of EV types and species from the stool specimens obtained from captive and wild-living apes and monkeys in Cameroon. We found that captive chimpanzees and gorillas were primarily infected by EV types having a worldwide distribution in humans, thus confirming that close contacts can promote cross-species transmission of EVs between humans and NHP. We also found proposed new EV types infecting wild NHP that were genetically distinct from all known EVs. Given the potential cross-species transmission of at least some EV types and the increasing contacts between human and NHP, EVs infecting NHP could serve as a source of new emerging human viral pathogens with unpredictable symptomatology.

## Introduction

Enteroviruses (EVs), in the *Picornaviridae* family, form a diversified genus infecting numerous mammalian species including humans, Non-Human Primates (NHP), sheeps, cows and pigs [1], [2]. Most EV infections are asymptomatic, but they can cause a wide range of diseases that can be severe and occasionally fatal in humans and animals [1], [3], [4]. Human EVs can cause symptoms ranging from mild febrile illness to severe forms, such as common cold, gastroenteritis, upper and lower respiratory diseases, acute hemorrhagic conjunctivitis, aseptic meningitis, myocarditis, encephalitis, myelitis and acute flaccid paralysis (AFP) [5]. An efficient surveillance system of AFP has been set up in the world thanks to the global poliomyelitis eradication program. Apart from AFP, other enteroviral diseases syndromes are rarely taken into consideration in the developing world including in sub-Saharan Africa despite the evidence of extensive EV circulation [6]–[9].

EVs are small non-enveloped viruses having a capsid with icosahedral symetry. Their genome is made up of a single poly-adenylated positive RNA strand of about 7.5 kb, which is covalently linked to a small viral protein VPg at the 5′ terminus. The single open-reading frame (ORF) is flanked by two untranslated regions (5′UTR and 3′UTR). The large polyprotein translated from the genomic RNA strand is cleaved into four structural proteins (VP1–VP4) and the non-structural proteins.

The Executive Committee of the International Committee on the Taxonomy of Viruses (ICTV) approved on February 2013 to rename the EV species without their host names. The *Enterovirus* genus now comprises 12 species: EV-A to -D (former *Human Enterovirus A* [HEV-A] to -D), *Rhinovirus A* [RV-A] to -C (former *Human Rhinovirus A* [HRV-A] to -C), EV-E and -F (former *Bovine Enterovirus 1* [BEV-1] and -2), EV-G (former *Porcine Enterovirus B* [PEV-B]), EV-H (former *Simian Enterovirus A* [SEV-A]) and EV-J (gathering previously unclassified simian viruses (http://ictvonline.org/virusTaxonomy.asp?version=2012).

Even though EVs are ubiquitous in humans, it is noteworthy that the worldwide distribution of EV types and species is not homogenous. Some human EV types and variants are more prevalent or geographically restricted in certain parts of the world. For instance, some EV-A such as EV-A71, Coxsackievirus A6 (CV-A6) and CV-A16 are involved in large epidemics primarily in Asia. EV-B species are seemingly predominant in temperate countries [10]–[12] even though the application of molecular characterization directly on clinical samples (without cell culture techniques) has recently uncovered several new EV-C types [13]–[17]. High rates of EV-C types have been repeatedly reported in tropical countries [7], [8], [18], [19]. Our recent studies revealed an extensive circulation with a tremendous genetic diversity of EVs in humans living in Cameroon as well as in the Central African Republic. A remarkable number of human EVs belonged to phylogenetic lineages seemingly specific to sub-Saharan Africa: a new EV-A71 genogroup E, EV-C105, EV-B93, EV-D94, EV-D111, EV-D120 as well as several variants of the types CV-A13 and E-7 [8], [14], [18], [20], [21].

In contrast to human EVs which are extensively studied, simian EVs (NHP EVs) have been hardly considered. Most NHP EVs were primarily isolated in the 1950s to 1970s from primary cell cultures or clinical specimens originating from captive or wild-caught primates used in biomedical research [22]–[29]. The molecular characterization of 19 historical simian virus types thought to be EVs revealed that only 13 of them were actually members of the *Enterovirus* genus whereas the others defined a novel picornavirus genus termed *Sapelovirus* [30]–[32]. Seven of the confirmed simian EV types shared the same species with human-derived EV types: BaEV, SV19, SV26, SV35, SV43 and SV46 belonged to the EV-A species while SA5 belonged to the EV-B species. Six other simian EV types were more distantly related to those of human origin and were thus classified as separate species, SEV-A (A2plaque, SV4, and SV28) and EV-J (SV6, N125 and N203) respectively [30], [33].

As other central African countries, Cameroon houses a wide range of Old World primates' species including apes (chimpanzees and gorillas) and at least 28 species of Old World monkeys. A recent study of EVs infecting wild apes in Cameroon identified 3 virus types including EV-A76 and EV-D111 [34] which also circulate among humans in central Africa [8], [18], [21]. However, the circulation and genetic diversity of EVs infecting African NHP is still poorly documented.

We further investigated the genetic diversity of EV types and species in the stool specimens originating from captive and wild-living apes and monkeys in Cameroon. We found virus types known to infect humans primarily in captive chimpanzees and gorillas but also in wild chimpanzees. In addition, some EVs from wild monkeys and chimpanzees were genetically distinct from all existing virus types, thus indicating that the overall picornavirus landscape is much more diversified than previously suspected.

## Materials and Methods

### Samples

Stool specimens of NHP were collected with the permission of the Cameroonian Ministries of Health, Environment and Forestry, and Research. Stool specimens of captive apes and monkeys were collected in April 2008, in a single day, at the Mvog-Beti zoo in Yaounde and the apes' sanctuary in Mfou (a rural village located at about 40 Km from Yaounde). Captive NHP were primarily wild-born orphaned animals living mainly in Southern rain forest areas of Cameroon. These animals have been brought to the zoos or sanctuaries, generally after their mother had been killed by hunters. About 5 g of stool specimens collected from freshly deposited stools were transported in refrigerated containers and kept frozen at −20°C until use. Further stool specimens originating from wild NHP were collected between June 2006 and October 2008 at forest sites located in the southern part of Cameroon. These specimens were part of a larger stool collection constituted for SIV screening [35], [36]. About 2 g of stool specimen was collected from freshly deposited dung in a cryotube. These tubes were kept frozen in nitrogen containers for about 3 weeks and subsequently transported to the laboratory for storage at −20°C.

The preparation and clarification of stool suspensions at 20% (Wt/Vol) were performed as described elsewhere [37].

### Fecal nucleic acids extraction

The fecal RNAs were extracted and purified from 140 µL of clarified stool suspensions using the QIAamp Viral RNA Mini Kit according to the manufacturer's instructions (Qiagen, France). RNAs were eluted in 60 µL of nuclease-free water.

Fecal DNAs were extracted from 220 mg of EV-positive stool samples using the QIAamp DNA Stool Mini kit (Qiagen, France), according to the manufacturer's instructions. DNAs were eluted with nuclease-free water in a final volume of 100 µL DNA solution.

Total RNAs and DNAs were stored at −80°C until use.

### Amplification of enterovirus genome from fecal RNAs

Purified RNAs were tested for EVs using a VP1-specific semi-nested RT-PCR (RT-snPCR) as previously described [38], [39].

All RNA samples containing EVs detected with the VP1-specific amplification, including weak signals, insufficient for amplicon sequencing, were also subjected to RT-PCR targeting 5′UTR and 3D^pol^ regions. In the 5′UTR region, a 450 base pairs DNA fragment was amplified with the primer pair UG52/UC53 [40]. RT-PCR products were then used as templates in semi-nested PCR using the PCR forward primer UG52 and a new reverse primer (AS3: 5′-ACGGACACCCAAAGTAGTCGGT-3′, nt 559 to 538 relative to SV46), designed from a highly conserved region in the alignment of all database available 5′UTR sequences of simian EVs and representative human EVs. A portion of the 3D^pol^ region was amplified with primers and reaction conditions previously reported [38], [41].

In order to refine virus type identification, complete VP1 sequence was determined for a set of viruses selected as representatives of all lineages revealed by the analysis of partial VP1 sequences. Overlapping amplicons encompassing the VP3-2A region were amplified by PCR with a combination of consensus degenerate primers (AMTH, 224, 011 and GDCL) [39], [41], [42] and semi-nested PCR carried out with strain-specific forward and reverse primers.

### Species determinations and search for prospective human-derived biological contaminations

For all stool specimens positive for EV detection and/or isolation, primate species were determined by mitochondrial DNA (mtDNA) analysis as described elsewhere [35], [43], [44].

In addition, in order to verify that EVs detected in NHP samples did not result from prospective contaminations by human-derived biological materials, fecal DNA from all EV-positive samples was screened for human DNA by using the primers pair FOXP2-humF/FOXP2-commonR [45]. This sensitive PCR can specifically amplify a portion of the forkhead box P2 (FOXP2) gene of the human DNA in a mixture containing DNA from other vertebrates, including chimpanzees and gorillas. Human DNA was extracted from a stool sample and used as positive control to validate that the sensitivity of the amplification was as previously reported [45].

### Sequencing of amplicons and sequence analysis

Specific PCR products were either directly purified with QIAquick PCR Purification kit or gel-isolated and purified using the QIAquick Gel Extraction kit (Qiagen, France) following the manufacturer's protocol. Amplicons were subjected to direct sequencing using the BigDye terminator v3.1 kit (Applied Biosystems) and an ABI Prism 3140 automated sequencer (Applied Biosystems). They were sequenced in both directions using amplification primers.

Partial or complete VP1 capsid coding gene of each virus was pairwise compared with the prototype sequences of all known human and simian EV prototype strains retrieved from databases (http://www.picornaviridae.com/sequences/sequences.htm). Field isolates were considered to belong to a given type when they featured a nt identity ≥75% and an aa identity ≥88% with the prototype strain of this type [46].

Full-length VP1 sequences of viruses that were noticeably divergent from any known EVs (nt identity<75%), were submitted to the *Picornaviridae* Study Group of the International Committee for the Taxonomy of Viruses for assignment as a new type.

Multiple sequence alignments were performed with CLC Main Workbench 5.7.2 software (CLC bio, Aarhus, Denmark). Phylograms were inferred both by the distance and maximum likelihood methods implemented in MEGA, version 5.10 [47] and PhyML 3.0 [48] respectively. Distance based phylogenetic trees were reconstructed by the neighbor-joining method with the Kimura two-parameter method for computing evolutionary distances [49]. All alignment gaps were removed from the analysis for each sequence pair. Maximum-likelihood (ML) algorithm was implemented under the HKY85 model of substitutions [50] with a transition/transversion ratio of 8.0. The reliability of tree topologies was estimated by bootstrap analysis with 1,000 pseudoreplicate data sets.

Nucleotide sequences generated in this study were submitted to databases (accession numbers KF614472-KF614507 for partial 5′UTR sequences, KF541625-KF541633 for partial VP1 sequences, KF541634-KF541649 and KF700264-KF700265 for full-length VP1 sequences, and KF648600-KF648622 for partial 3D^pol^ sequences).

### Cell lines and virus isolation

Stool samples from wild NHP that were positive for EV molecular detection and all samples from captive NHP were tested for virus isolation by cell cultures. A volume of 0.2 mL of chloroform-treated and clarified stool suspensions were inoculated onto monolayered Human rhabdomyosarcoma (RD), human larynx epidermoid carcinoma (HEp-2c), African Green monkey kidney (Vero), and rhesus monkey Kidney (LLC-MK2) cells. RD, HEp-2c and Vero cells were maintained at 36°C in D-MEM medium while LLC-MK2 cells were maintained at 36°C in the 199 medium. Both media were supplemented with 2% foetal calf serum and 2 mM L-glutamine. Infected tubes were microscopically checked for 7 days to detect the appearance of cytopathogenic effects (CPE). The supernatant of the cultures which had remained negative were frozen, thawed, and inoculated to fresh cell cultures. Then newly inoculated cultures were checked for the next 7 days. Virus isolates harvested from cell cultures were analyzed using the molecular approaches described above.

## Results

Overall, 615 stool specimens collected from NHP in Cameroon were tested in this study. A total of 516 specimens originated from wild-living NHP including 96 chimpanzees, 403 gorillas and 17 monkeys. Further 99 stool specimens were collected from captive NHP housed at the Mvog-beti zoo (20 monkeys) and the Mfou sanctuary (42 chimpanzees, 17 gorillas and 20 monkeys) (Table 1). RT-snPCR targeting the capsid VP1 coding region detected EV RNA in 33 out of 615 NHP stool samples (5.4%) with the rates of 15.2% (15/99) and 3.5% (18/516) in captive and wild NHP, respectively (Table 1).

**Table 1:** Global description of the primates samples and overall PCR, isolation and VP1 sequencing results.

	Species	n	PCR Pos N (%)	Isolation (PCR-positive samples) N	Isolation (PCR-negative samples) N^a^	combined PCR and isolation N (%)	VP1 sequencing N^b^
Wild	Chimpanzees	96	10 (10.4)	0	ND	10 (10.4)	6
	gorillas	403	1 (0.25)	0	ND	1 (0.25)	1
	monkeys	17	7 (41.2)	0	ND	7 (41.2)	4
**Total**		**516**	**18 (3.5)**	**0**	n/a	**18 (3.5)**	**11**
Captive	Chimpanzees	42	6^c^ (14.3)	1	3	9 (21.4)	9
	gorillas	17	3^d^ (17.6)	0	1	4 (23.5)	5
	monkeys	40	6 (15.0)	1	1	7 (17.5)	2
**Total**		**99**	**15 (15.2)**	**2**	**5**	**20 (20.2)**	**16**
**Grand Total**		**615**	**33 (5.4)**	**2**	n/a	**38 (6.2)**	**27**

a , ND, not done; n/a, not applicable.

b , Overall 12 VP1 amplicons showed very low signal insufficient for sequencing reactions. However, 11 of the corresponding viruses could be sequenced in the 5′UTR region while the remaining one was refractory to 5′UTR amplification but showed a divergent 3D^pol^ sequence (GenBank n° KF648605 and KF648607).

c , Includes one sample with a mixture of CV-A13 and an EV-A.

d , Includes one sample with a mixture of EV-A71 and EV-B82.

To increase the virus detection rate and to further characterize the viruses detected, all stool samples from captive NHP and stool samples from wild NHP that were EV-positive in molecular screening were inoculated to the cultures of RD, HEp-2c, Vero and LLC-MK2 cell lines. Virus isolation produced 7 isolates propagated on one or two of the cell lines used (Tables 1 and 2). All these isolates originated from captive NHP, five of which were negative using RT-snPCR targeting both 5′UTR and VP1 genomic regions. The final rate of EV detection/isolation among captive NHP was thus 20.2% (20/99) (Table 1).

**Table 2:** Identification of enterovirus types and species in captive and wild non-human primates according to their species.

Viruses^a^	Primate Hosts		Isolation	Highest identity score^c^ (%)			Identification^d^		GenBank accession no.
	Species^b^	living setting		prototype	nt	aa	virus type	virus species	
Chimpanzees									
CHB2*	*Pan t. troglodytes*	wild		EV-A76	84.5	93.0	EV-A76	EV-A	KF541633
CHB5*	*Pan t. troglodytes*	wild		EV-A76	86.2	96.0	EV-A76	EV-A	KF541631
CHB6	*Pan t. troglodytes*	wild		EV-A76	85.6	97.0	EV-A76	EV-A	KF541649
CHB7	*Pan t. troglodytes*	wild		EV-A76	85.3	94.9	EV-A76	EV-A	KF541648
CHB8*	*Pan t. troglodytes*	wild		EV-A76	85.9	95.0	EV-A76	EV-A	KF541632
CHE20	*Pan t. troglodytes*	wild		EV-J103	61.1	66.4	**EV-J121**	EV-J	KF541639
Gorillas									
GOJ01*	*Gorilla gorilla*	wild		EV-A92	68.8	69.0	potential new type	EV-A	KF541630
Monkeys									
RCMH03	red-capped mangabey	wild		SV6	57.8	52.2	**EV-123**	candidate new	KF541637
RCMH05	red-capped mangabey	wild		SV6	55.6	53.2	**EV-122**	candidate new	KF541636
RCMH06	Red-capped mangabey	wild		SV6	56.6	53.2	**EV-122**	candidate new	KF541638
RCMH07	*Mandrillus sphinx*	wild		SV6	57.9	52.2	**EV-123**	candidate new	KF541635
Chimpanzees									
Z004	chimpanzee	captive	HEp-2c	CV-A13	73.0	87.7	CV-A13	EV-C	KF541646
Z033*	*Pan t. troglodytes*	captive		CV-A13	69.0	87.3	CV-A13	EV-C	KF541627
Z034*	*Pan t. troglodytes*	captive		CV-A13	69.3	87.3	CV-A13	EV-C	KF541628
Z035*	*Pan t. troglodytes*	captive		CV-A13	69.3	87.3	CV-A13	EV-C	KF541629
Z036	*Pan t. troglodytes*	captive	HEp-2c	CV-A13	73.3	87.7	CV-A13	EV-C	KF541645
Z108-1&	*Pan t. troglodytes*	captive	HEp-2c	CV-A13	73.3	87.7	CV-A13	EV-C	KF541647
Z046	*Pan t. troglodytes*	captive		EV-A119	95.7	98.7	EV-A119	EV-A	KF541634
Z055	*Pan t. troglodytes*	captive	RD	E-29	80.6	95.9	E-29	EV-B	KF541644
Z110*	*Pan t. troglodytes*	captive		E-15	79.8	95.4	E-15	EV-B	KF541626
Gorillas									
Z052	*Gorilla gorilla*	captive		CV-A24	78.3	89.5	CV-A24	EV-C	KF541640
Z097	*Gorilla gorilla*	captive	HEp-2c	E-15	78.9	90.8	E-15	EV-B	KF541641
Z098-1*&	*Gorilla gorilla*	captive		EV-B82	81.6	91.5	EV-B82	EV-B	KF541625
Z098-2&	*Gorilla gorilla*	captive		EV-A71	79.4	94.3	EV-A71	EV-A	KF700265
Z099	*Gorilla gorilla*	captive		EV-A71	79.4	94.3	EV-A71	EV-A	KF700264
Monkeys									
Z057	*Mandrillus sphinx*	captive	HEp-2c and Vero	SA5	82.1	92.9	SA5	EV-B	KF541643
Z088	*Mandrillus sphinx*	captive	HEp-2c and RD	E-29	80.5	95.6	E-29	EV-B	KF541642

a Viruses from wild primates are abbreviated in the following format: two letters code standing for the primate species identified in the field (CH, chimpanzee; GO, gorilla; RCM, red-capped mangabey), followed by one later (from B to J) corresponding to the round of sample collection on the field and the serial number of the stool sample. Viruses from captive primates are abbreviated using the letter “Z” standing for Zoo, followed by the serial number of the stool sample. For samples containing 2 viruses, “−1” and “−2” were used to distinguish among each others. Viruses specified with an asterisk (*) have been typed using partial VP1 sequences.

b Species designated with scientific names were confirmed by the analysis of a portion of simian mitochondrial DNA sequences; *Pan t. troglodytes*, *Pan troglodytes troglodytes*.

c nt, nucleotides; aa, amino acids ; SA5, simian picornavirus 17.

d Candidates new types discovered in this study are highlighted in bold.

& Chimpanzee sample Z108 contained two isolates Z108-1 (CV-A13) and a potentially divergent EV-A (Z108-2) which could be sequenced only in the 3D^pol^ region; the gorilla sample Z098 contained two isolates Z098-1 (EV-B82) and Z098-2 (EV-A71).

In this study, the PCR based screening for human DNA was performed on all EV-positive samples regardless of the virus type identified. In contrast to the positive control (a human stool sample), none of the EV-positive stool samples, including those containing virus types previously reported in humans, showed a PCR-based evidence of contamination by human-derived tissues.

### Molecular typing

Partial or complete VP1 sequence could be successfully determined for 27 EVs detected and/or isolated (Table 1). VP1 sequences determined from isolates Z057 and Z108-1 were identical to those obtained directly from viral RNA extracted from corresponding fecal samples. Overall VP1 amplicons from 12 samples showed very low signal insufficient for sequencing reactions. However, the viruses from 11 of these samples could be sequenced in the 5′UTR region while the one from the remaining sample was refractory to 5′UTR amplification and was shown to have a divergent 3D^pol^ sequence (GenBank n° KF648607) (Table S1).

#### Apes derived enteroviruses

Of the 21 apes-derived EVs identified, 13 from chimpanzees and 5 from gorillas belonged to virus types previously reported in humans. These known human EVs belonged to the species EV-A (5 EV-A76 and 2 EV-A71), EV-B (2 E-15, 1 E-29 and 1 EV-B82), and EV-C (6 CV-A13 and 1 CV-A24) (Table 2). Apart from E-15 and EV-B82, all these EV types have been previously identified among humans in our recent study in Cameroun [8]. For the first time, EV-A71, which is known to have a high potential to cause severe disease outbreak in humans [51], [52], was found in captive NHP-derived samples. Interestingly EV-A76, a known human EV type, was found in wild chimpanzees, in accordance with a recent study reporting EV-A76 in a wild chimpanzee in Cameroon [34].

Besides the EV types well known in humans, recently discovered virus types as well as proposed new virus types were identified in chimpanzees. The full-length VP1 sequence of the virus Z046 (captive chimpanzee) enabled its unambiguous assignment as an EV-A119, an EV type recently discovered in a human in Cameroon [6]. The virus CHE20 displayed sequence similarities lower than 75% and 85% with all databases available field and prototype strains at nucleotide (nt) and aminoacid (aa) levels respectively (Table 2). The *Picornaviridae* Study Group (PSG) of the International Committee for the Taxonomy of Viruses proposed the assignment of the virus CHE20 (wild chimpanzee) into a new type, designated EV-J121 (http://WWW.picornastudygroup.com/types/enterovirus/ev_types.htm). The partial VP1 sequence of the unique virus from a wild gorilla GOJ01 was somewhat related to the species EV-A. However, it was quite divergent from all known human and simian EVs. Further analysis is required to draw a conclusion about the type assignment of this potential new EV-A.

Two samples contained a mixture of 2 viruses: CVA-13 and a potentially divergent EV (which could be sequenced only in the 3D^pol^ region) originating from a captive chimpanzee sample (Z108); EV-A71 and EV-B82 originating from a captive gorilla sample (Z098) (Table 2 and Table S1).

#### Monkeys-derived enteroviruses

Among the 6 monkey EVs that could be typed, the virus Z088 from a captive mandrill was an E-29, a virus type circulating among humans in Cameroon [8] and elsewhere. The five other monkeys' viruses were clearly distinct from EV types isolated in humans. The virus Z057, originating from a captive mandrill, was a simian picornavirus 17 whose prototype strain SA5 was originally recovered from a vervet monkey, native to Africa. All remaining 4 viruses from wild monkeys did not reach the sequence similarity thresholds (75% nt and 85% aa identities) allowing their classification into existing virus types. Indeed, related viruses RCMH5 and RCMH6 on one hand, RCMH3 and RCMH7 on the other hand, were distinct to all prototype strains and were thus registered as proposed new EV types, designated EV-122 and EV-123 respectively (Table 2).

### Phylogenetic relationships of the NHP enteroviruses

Since phylograms inferred using Maximum Likelihood (ML) and Neighbor Joining (NJ) algorithms displayed very similar patterns, only NJ trees were considered.

**Figure 1:**
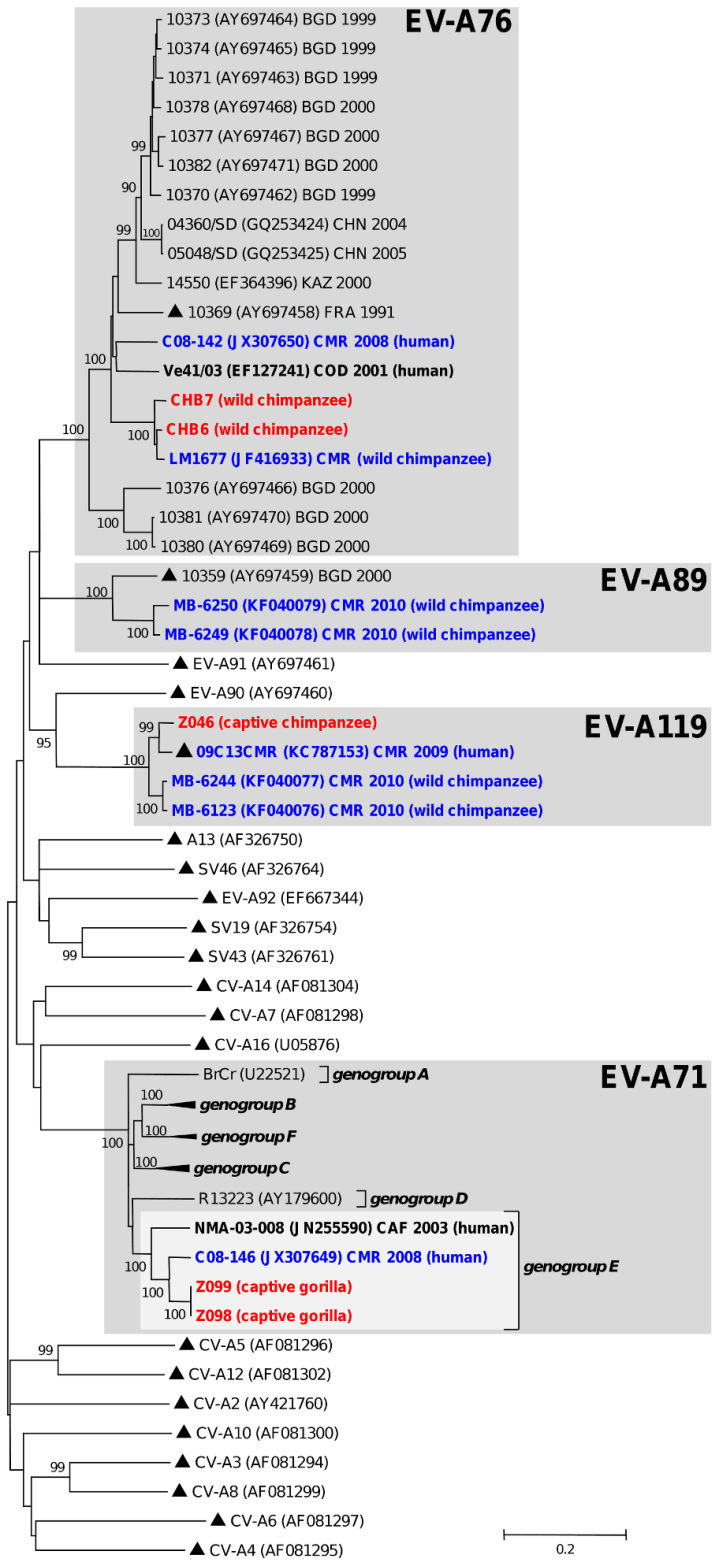
Phylogenetic relationships among *Enterovirus A* strains from human and non-human primates. The NJ tree is based on the alignment of the full-length VP1 sequences. Viruses of non-human primates' origin are highlighted in bold red with reference to the host names in brackets. The year and country of isolation are indicated for EV-A71, EV-A76, EV-A89 and EV-A119, if known (BGD, Bangladesh; CAF, Central African Republic; CHN, China; CMR, Cameroon; COD, Democratic Republic of the Congo; FRA, France and KAZ, Kazakhstan). Strains previously reported in Cameroon (bold blue) and other central African countries (bold black) are indicated with host species in brackets. Prototype strains are highlighted by triangles (▴). The scale is shown at the bottom as substitutions per site. Viruses belonging to enterovirus species commented in the text are gathered in grey-shaded boxes.

**Figure 2:**
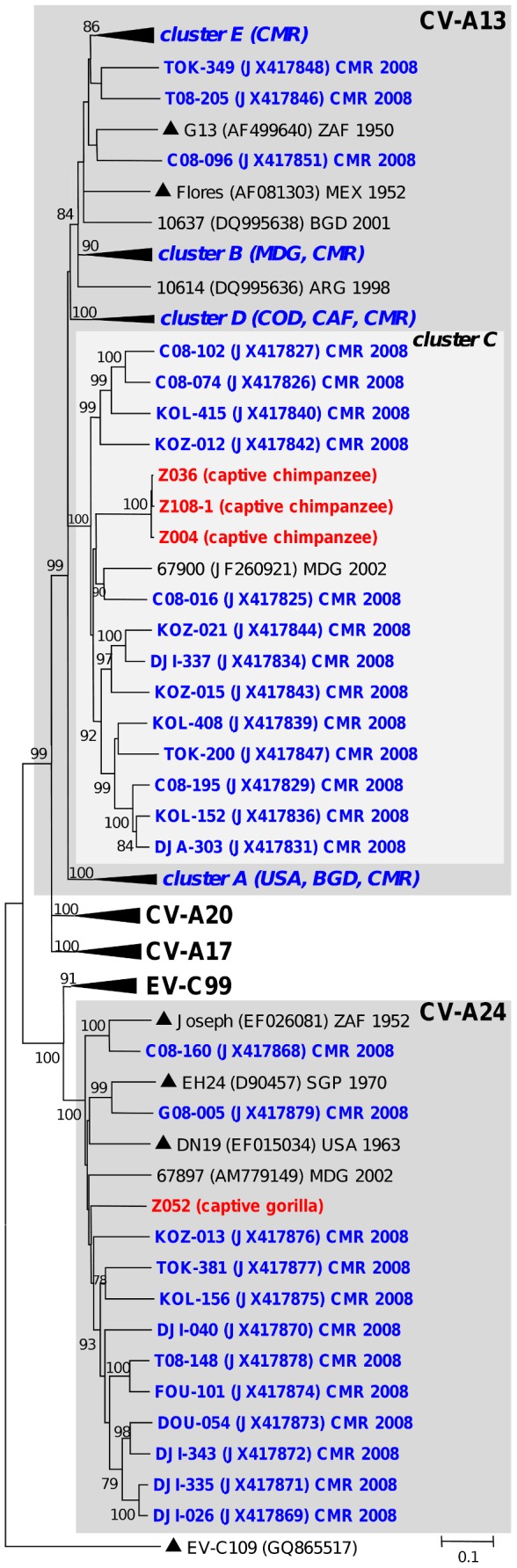
Phylogenetic relationships among *Enterovirus C* strains from human and non-human primates. The NJ tree is based on the alignment of the full-length VP1 sequences. Studied viruses from apes are highlighted in bold red with host names indicated in brackets while human-derived isolates from Cameroon are specified in bold blue. Prototype strains are indicated by triangles (▴). For clarity, type and lineage-specific clusters containing exclusively human isolates have been collapsed. The scale is shown at the bottom as substitutions per site. Viral isolates belonging to enterovirus species commented in the text are gathered in grey-shaded boxes.

Some NHP viruses featured close genetic relationships with EVs which have been already shown to circulate in this region. This was exemplified by chimpanzees-derived EV-A76 which displayed a close relationship with another chimpanzee strain LM1677 recently detected in a Cameroonian wild chimpanzee [34]. Both viruses formed together a chimpanzee-specific group (bootstrap value of 100%) that was somewhat distinct to human-derived EV-A76 including those from Cameroon (CMR) and the Democratic Republic of Congo (COD) (Figure 1). This group also included other closely related EV-A76 (CHB2, CHB5 and CHB8) detected in chimpanzees' samples (not shown in figure 1 because only partial VP1 sequences were available for these viruses - see table 2). In the same manner, EV-A119 from a captive chimpanzee grouped reliably with the homotypic prototype recently identified in a human in Cameroon [6] (Figure 1). Two other EV-A119 full-length VP1 sequences from a previous study originated from wild chimpanzees and a gorilla stool samples collected in Cameroon in 2010 [20] (Figure 1). Gorillas-derived EV-A71 fell into EV-A71 genogroup E, recently identified in sub-Saharan Africa [8], [18], [53] (Figure 1). Similar clustering pattern was displayed by chimpanzees' CV-A13 (Figure 2). All six partial and complete CV-A13 VP1 sequences defined a unique phylogenetic sub-cluster (98.5 to 100.0% nt identity between each other) gathering viruses originating from the same enclosure in the sanctuary of Mfou (data not shown). These viruses fell reliably within the CV-A13 cluster C gathering isolates recovered from humans in Cameroon and Madagascar from 2002 to 2009 [8], [18], [54] (Figure 2).

**Figure 3:**
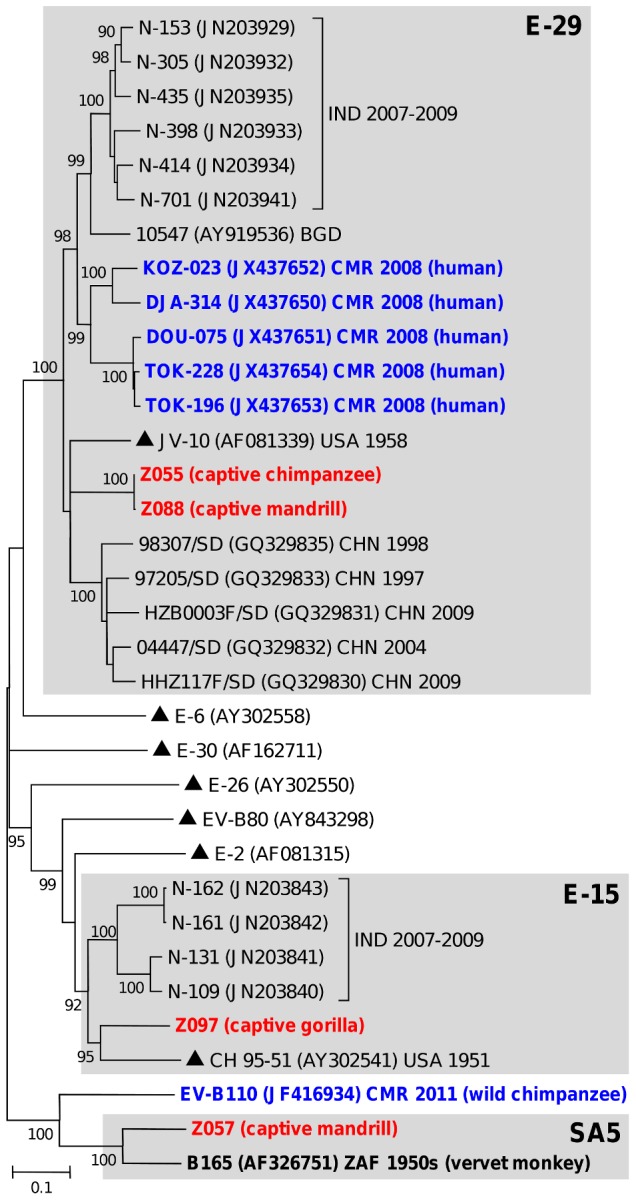
Phylogenetic relationships among *Enterovirus B* strains from human and non-human primates. The NJ tree is based on the alignment of the full-length VP1 sequences. Studied viruses from non-human primates are highlighted in bold red with the host names specified in brackets. Other human and simian viruses detected in Cameroon are in bold blue. The year and country of isolation are indicated for some viruses (BGD, Bangladesh; CHN, China; CMR, Cameroon; IND, India; USA, United States of America; ZAF, South Africa). Prototype strains are indicated by triangles (▴). Scale is shown at the bottom as substitutions per site. Viral isolates belonging to enterovirus species commented in the text are gathered in grey-shaded boxes.

In contrast, a number of EVs from NHP did not show a clear relatedness with viruses already known to circulate in central and west Africa or elsewhere. For instance, the gorilla-derived CV-A24 was somewhat distinct from isolates originating from Cameroon and did not display peculiar relatedness with any of the known African lineages of CV-A24 [8] (Figure 2). Similarly, the chimpanzee and mandrill-derived E-29 were highly similar to each other but differed significantly from their counterparts originating from humans in Cameroon (Figure 3).

There were no E-15 and SA5 isolate identified so far in humans and NHP in the considered region. The E-15 isolate Z097 from a captive gorilla was quite distinct from homotypic strains infecting humans (Figure 3). The mandrill's simian picornavirus 17 Z057 was distinct from the wild chimpanzee EV-B110 from Cameroon and clearly belonged to the SA5 type originally described in a vervet monkey, native to Africa (Figure 3). This provided further support to the fact that at least some EV types could naturally infect different NHP species.

**Figure 4:**
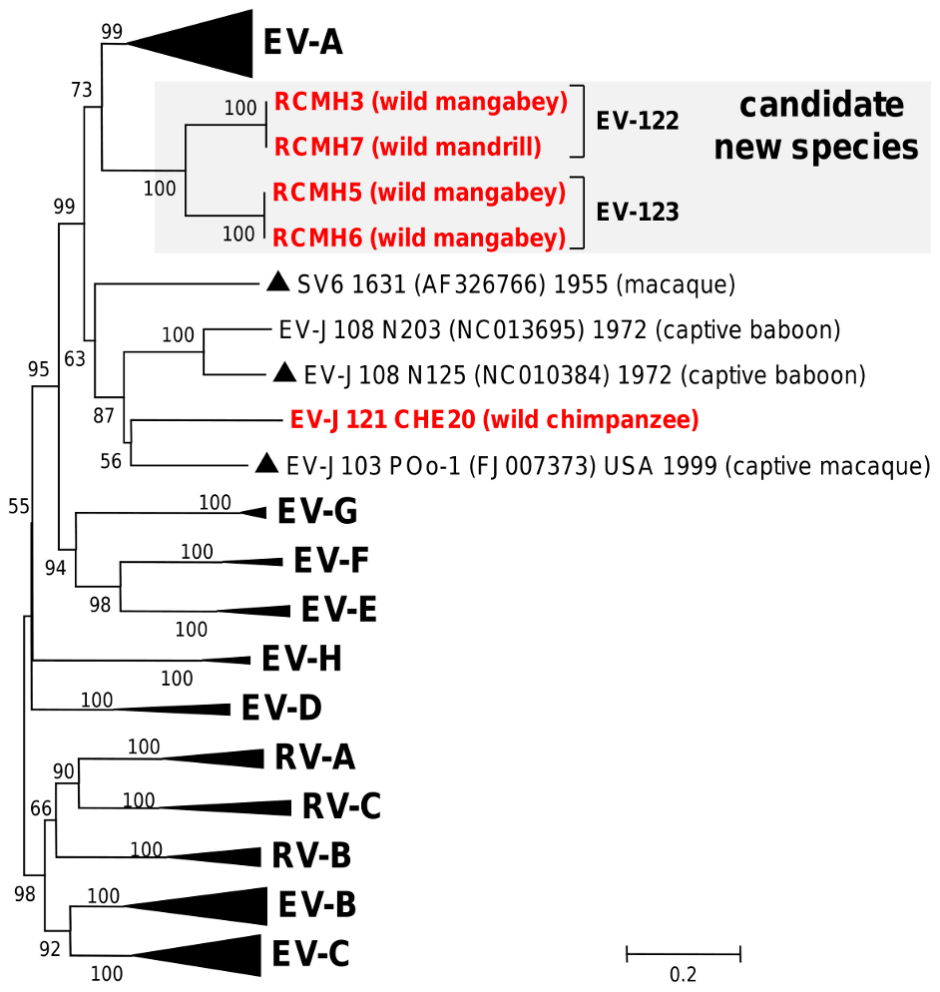
Phylogenetic relationships among the candidate new enterovirus types and species and other known human and animal enteroviruses. Studied viruses from non-human primates are highlighted in bold red with host names indicated in brackets. For clarity most species clusters have been collapsed. The scale is shown at the bottom as substitutions per site.

This study also identified hitherto unknown virus types that were phylogenetically distinct from all known human and animal EVs. In accordance with pairwise comparison with prototype strains, RCMH3/RCMH7 and RCMH5/RCMH6 from wild monkeys did not fit into any of the existing EV types and species. They defined two proposed new EV types: EV-122 and EV-123 (Figure 4). With only 55.6 to 57.9% (nt) and 52.2 to 53.2% (aa) VP1 sequence identities to the closest EV type SV6 [30], EV-122 and EV-123 clearly defined a candidate new EV species. This proposed new EV species was interspersed between EV-A and EV-J (Figure 4). Compared to other EV species, EV-A, EV-J and the candidate new species constituted a separate branch supported by a bootstrap value of 99% (Figure 4). The wild chimpanzee virus CHE20 was strikingly divergent compared to EVs originating from humans and chimpanzees. It grouped reliably with previously described types SV6, EV-J103 and EV-J108 (Figure 4). Within the EV-J cluster, the virus CHE20 defined a new type designated EV-J121. It was more closely related to EV-J103 POo-1 (nt & aa identity of 62.2% and 66.4%, respectively - Table 3) than to the other EV-J types (nt & aa identity <58.7 & 58.8%, respectively). However, the similarity scores between EV-J121 CHE20 and EV-J103 POo-1 VP1 sequences were very heterogeneous along the whole VP1 genomic region (Figure S1). In the 5′ two thirds of the VP1 region (nt 1–591, according to CHE20 numbering), sequences featured high nt and aa identities of 65.8% and 66.4%, respectively. In addition these CHE20 and POo-1 sequences gathered in the same branch of a phylogenetic tree supported by a high bootstrap value (100% - Figure S1). In contrast sequences of the last one third of the VP1 region (nt 592–867) showed low nt and aa identities (54.3% and 55.4%, respectively) and fell in separate branches (Figure S1). These findings suggested that EV-J121 VP1 region may have resulted from a recombination event between two distinct viruses.

**Table 3:** VP1 sequence relationships between the newly sequenced EV-J121 and other currently recognized EV-J strains.

	% identity (nucleotide/amino acids)			
EV-J strains	SV6 1631	EV-108 N203	EV-108 N125	EV-121 CHE20
EV-J108 N203	58.1/52.4			
EV-J108 N125	57.1/48.9	79.7/89.7		
EV-J121 CHE20	55.0/49.7	58.3/58.4	58.7/58.8	
EV-J103 PO0-1	57.0/53.4	59.8/63.2	59.9/61.2	62.2/66.4

### Comparative analysis of non-structural genomic sequences of EVs from NHP and humans

**Figure 5:**
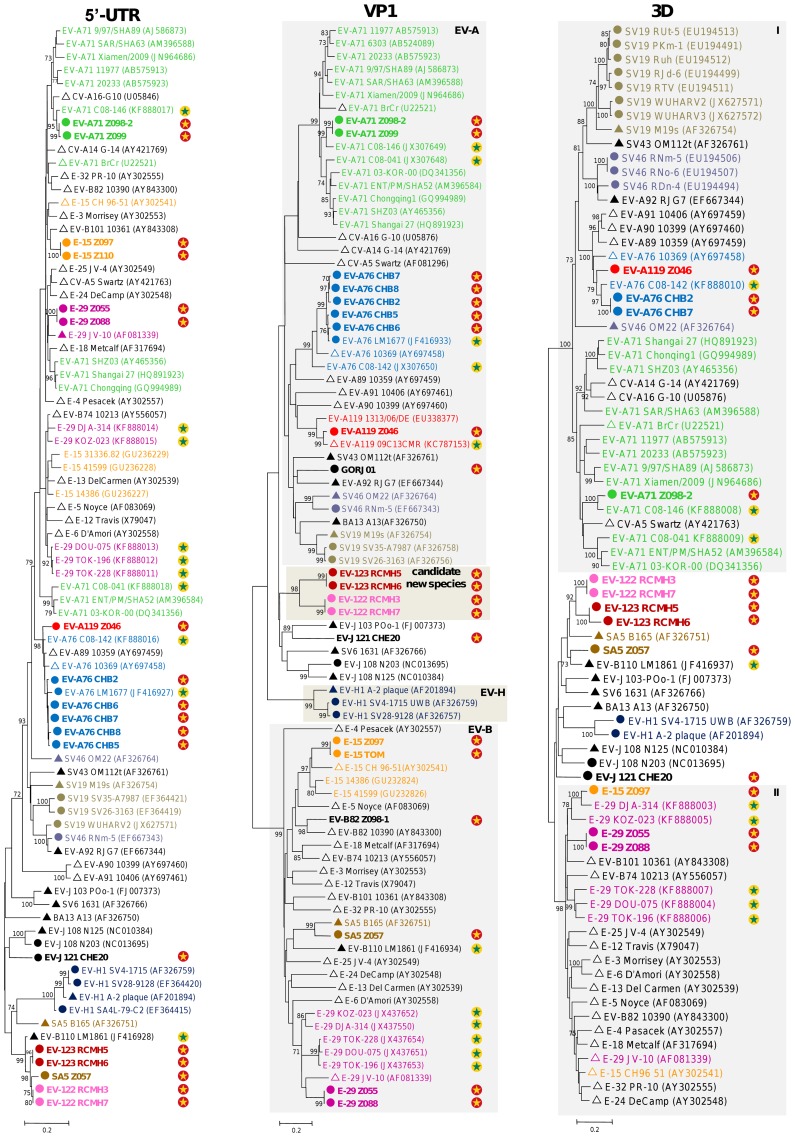
Phylogenetic relationships among the 5′UTR, VP1 and 3D^pol^ sequences of enteroviruses from human and non-human primates. Only non species C enteroviruses were considered in these trees. The 5′UTR, VP1 and 3D^pol^ trees were based on the alignments of partial sequences (positions according to EV-A71 strain BrCr numbering: 193–430 for 5′UTR, 2615–2903 for VP1 and 6034–6377 for 3D^pol^ regions). Only non species C enteroviruses whose partial or complete VP1 sequences could be generated were considered in this comparative analysis. Prototypes strains originating from non-human primates (▴) or humans (Δ) are indicated. All other non-human primates strains from previous studies are distinguished by black circles (•) and those from this study are color-coded according to virus types. Non-human primates-derived viruses characterized in this study are further highlighted by yellow stars whereas green stars specify viruses previously identified in Cameroon either from humans or non-human primates.

To analyze the genetic relationships between non-structural genomic regions of EV from NHP, sequences of the 5′UTR and 3D^pol^ regions of most studied viruses were compared with those of human origin from Cameroon (Figure 5).

Interestingly, the 5′UTR and 3D^pol^ sequences of the gorillas-derived EV-A71 featured a close relatedness with the human EV-A71 isolate C08-146 belonging to the African genogroup that was recently reported in Cameroon [8] (Figure 5). This indicates that both human and gorilla EV-A71 have evolved from a recent common ancestor without apparent recombination events involving the other considered EV strains.

All EV-A76 sequences determined in this study were closely related to those of previously reported human and simian EV-A76 from Cameroon (Figure 5). In addition, most EV-A76 sequences and in particular those of the 5′UTR and 3D^pol^ regions were closely related to those of the simian EV-A119 Z046 and to a certain extent to those of human-derived EV-A89, EV-A90 and EV-A91. This suggested that these EV-A types have evolved through circulation in both humans and NHP and may be through recombination among each others.

All other 5′UTR and 3D^pol^ sequences of NHP EVs clustered apart from those of homotypic viruses of human origin. This result corroborated those of the VP1 region and argued in favor of a relative specificity of NHP-derived EVs compared to their homotypic human-derived isolates (Figure 5). Similar results were found when the 5′UTR and 3D^pol^ sequences of NHP-derived CVA-13 and CVA-24 were compared to those of homotypic strains previously reported among humans in Cameroon [8] (data not shown).

Concerning the 3D^pol^ region in particular, most of the sequences grouped into two main clusters. The cluster I (bootstrap value of 100%) only contained EV-A sequences while the cluster II (bootstrap value of 98%) contained only sequences of EV-B. This clustering pattern is in accordance with previous observations suggesting that in a majority of cases, recombination events in the non-structural part of the genome involve virus types belonging to the same species. Nonetheless, sequences of types BA13 (EV-A species), SA5 and EV-B110 (EV-B species) were found not to belong to these two clusters and were more closely related to sequences from other species. Interestingly, all these EV-A and -B sequences falling outside the two EV-A and EV-B-specific 3D^pol^ clusters were of NHP origin. In particular, the 3D^pol^ (and the 5′UTR) sequences of some simian SA5 and EV-B110 (EV-B species) isolates grouped consistently with those of the novel NHP-derived EV-122 and EV-123, suggesting evolution through interspecies recombination.

## Discussion

The main goal of this study was to characterize the genetic diversity of EVs in both captive and wild NHP in Cameroon and to compare this diversity with that found in humans. Our findings revealed the co-circulation of some EVs in humans and NHP and uncovered proposed new EV types and species currently circulating in wild apes and monkeys.

All but one of the 16 EVs identified among captive NHP belonged to virus types that circulate among humans (Table 1), thus confirming that the NHP species are highly sensitive to human EVs and are at risk of infections with these pathogens. These results are similar to those documenting the presence of a wide range of human EVs in synanthropic NHP living in close association with humans in Bangladesh, 100% of the detected picornaviruses belonging to virus types reported in humans [55]. In contrast, in captive monkeys living in Bangladesh zoos only 15% of picornaviruses belonged to virus types reported in humans [56]. This last result may appear conflicting with our findings; however it could be explained by differences in captivity conditions for NHP in Bangladesh zoos and Cameroon sanctuaries. In Bangladesh, NHP were kept out of the way of humans in cages limiting physical contacts whereas in Cameroon most captive NHP were living in vast enclosures, with more easy interactions with employees and the public [56].

Among EVs of human origin detected in captive settings in Cameroon were Echoviruses and CV-A which have been shown to be worldwide distributed in humans. In particular CV-A13 and CV-A24 have been shown to have high endemicity and diversity among humans in central Africa [8], [18]. Human to NHP transmission may have occurred as previously reported in another context, in Bangladesh. However, a relative sequence divergence was featured by VP1 gene of NHP derived E-29 and CV-A24 compared to their homotypic human counterparts from Cameroon. This suggests that some EV types known in humans may have been co-circulating and evolving independently for a while in NHP. However, this observation may be biased by the fact that some human and NHP EVs considered for this comparison originated from distant regions in Cameroon. At this stage, it cannot be excluded that captive NHP EVs belonging to virus types circulating in humans originated from wild NHP.

Despite the fact that EV-A76 was described recently [57], it is well distributed in humans with high rates reported in southeastern Asia and central Africa [55], [57]–[59]. Strikingly, both central Africa and southeastern Asia, where EV-A76 is highly prevalent in humans, are homes to several endemic NHP species. Strengthening previous observations [34], our study identified EV-A76 in wild chimpanzees. Even though their human origin cannot be wiped out at this stage, it is likely these EV-A76 were indigenous to wild chimpanzees since they originated from stool samples collected in the remote jungle with minimal human contacts.

The identification of EV-A119 and EV-A71 genogroup E in captive apes and their apparent association to sub-Saharan Africa [6], [8], [9], [18] suggest their co-circulation in both human and NHP in this region. An apparent geographic association to sub-Saharan Africa has also been observed for EV-D111 which was originally found in a wild chimpanzee from Cameroon [34] and was subsequently shown to be distributed among humans in Central Africa [8], [18], [21]. These data suggest that NHP, especially apes, could serve as reservoirs of potential new EV types in humans.

Major discoveries of this study were that wild NHP were infected in most cases by typical simian EVs and that still unknown EV types and species were circulating in wild apes and monkeys. This indicated that either wild NHP are living out of the way of humans, with minimum physical contacts in Cameroon forests or, although less likely, that some EV types, species or genotypes are poorly adapted to humans. In addition, it is likely that the abundance and diversity of NHP EVs have been underestimated in this study, some EVs being refractory to PCR amplification using generic primers. In particular, wild gorilla showed a remarkably low EV detection rate at 0.25% (1/403). In accordance with this hypothesis, 5 samples of captive NHP that were PCR-negative in initial screening were shown to contain EVs that were successfully isolated in human cell cultures (RD and/or HEp-2c) (Table 1). Moreover, 12 samples yielding very low VP1 amplification signal could not be clearly identified. However, 7 of them showed 5′UTR sequences closely related to those of known human or NHP-derived EVs (data not shown). The remaining 5 samples contained viruses that showed quite divergent 5′UTR sequences (from a captive *Cercopithecus neglectus* and 3 wild chimpanzees) and 3D^pol^ sequences (from a wild chimpanzee), suggesting unknown EVs or picornaviruses (data not shown). The fact that some studied viruses were refractory to viral isolation or PCR amplification in one of the target genomic regions provided further support to the fact that our approach did not take exhaustively into account the actual diversity of EVs in NHP. However, as other studies, this one has demonstrated the existence of known and hitherto unknown EV types and variants in NHP [20], [34]. It is likely that hitherto unknown EVs remain to be discovered among NHP, in particular in the wild fauna. More powerful molecular approaches (like high throughput sequencing) will be helpful for an exhaustive assessment of the genetic diversity of EVs infecting NHP.

Although wild NHP were infected in most cases by typical simian EVs, it cannot be excluded that at times, these simian EVs could spread from NHP to humans living in adjacent habitats. Such transmission could happen directly from wild NHP to humans or could transit through the NHP populations kept in zoos and sanctuaries. These scenarios may have happened for a number of EVs discovered in humans and wild apes recently: EV-A76, EV-D111 and EV-A119 [6], [8], [18], [21], [57]. Wild NHP could play the role of reservoir and/or source of future emerging EVs with unpredictable symptomatology in humans. It is important to document the picornavirus landscape among NHP, especially in chimpanzees which are our closest primate relatives, in order to better predict the risks of their emergence and their pandemic potential in humans.

In Cameroon, wild chimpanzees have been shown to be infected with EVs related to those circulating in humans, suggesting possible cross-species transmission of EVs among primates (34). In the present study we showed that captive NHP of this country can be infected with well-known human EVs, as well as with typical simian EVs or EVs known to infect both humans and NHP. This supports the idea that zoos and sanctuaries may favor contacts and EV infection among humans and NHP. The finding of candidate new EV types and species as well as other potentially more divergent viruses revealed that the diversity of picornaviruses in NHP is broader than previously suspected. This argues for studying further EVs circulating in wild NHP and humans living in sympatry in order to estimate the exposure and potential dissemination of new EVs among humans in the forested regions in Cameroon.

## Supporting Information

Figure S1 **Comparative analysis of the full-length VP1 sequences of SV6, EV-J103, EV-J108, and the newly sequenced EV-J121 types.** The similarity plots were generated using SimPlot version 3.5.1 with 100 nucleotide (nt) windows, 20 nt increments, and the Kimura 2-parameter method with a transition-transversion ratio of 8.0. Partial VP1 based trees depicting the relationships between the strains considered are presented at the bottom of the corresponding target portion.
(TIF)

Table S1 **Summary of the detection and sequencing in the 5′UTR, VP1 and 3D^pol^ regions of enteroviruses among wild and captive non-human primates.**
(DOCX)

## References

[1] Pallansch MA, Roos R (2007) Enteroviruses: Polioviruses, Coxsackieviruses, Echoviruses, and Newer Enteroviruses. In: D. M. Knipe and P. M. Howley e, editor. Fields Virology. Philadelphia: Lippincott Williams and Wilkins. pp. 839–894.

[2] Boros A, Pankovics P, Knowles NJ, Reuter G (2012) Natural interspecies recombinant bovine/porcine enterovirus in sheep. J Gen Virol 93: 1941–1951. 22647375 10.1099/vir.0.041335-0

[3] Blas-Machado U, Saliki JT, Boileau MJ, Goens SD, Caseltine SL, et al (2007) Fatal ulcerative and hemorrhagic typhlocolitis in a pregnant heifer associated with natural bovine enterovirus type-1 infection. Vet Pathol 44: 110–115. 17197635 10.1354/vp.44-1-110

[4] Nielsen SC, Mourier T, Baandrup U, Soland TM, Bertelsen MF, et al (2012) Probable transmission of coxsackie B3 virus from human to chimpanzee, Denmark. Emerg Infect Dis 18: 1163–1165. 22709557 10.3201/eid1807.111689 PMC3376799

[5] Tapparel C, Siegrist F, Petty TJ, Kaiser L (2013) Picornavirus and enterovirus diversity with associated human diseases. Infect Genet Evol 14: 282–293. 23201849 10.1016/j.meegid.2012.10.016

[6] Ayukekbong J, Kabayiza JC, Lindh M, Nkuo-Akenji T, Tah F, et al (2013) Shift of Enterovirus species among children in Cameroon - Identification of a new enterovirus, EV-A119. J Clin Virol 58: 227–232. 23895932 10.1016/j.jcv.2013.07.005

[7] Rakoto-Andrianarivelo M, Rousset D, Razafindratsimandresy R, Chevaliez S, Guillot S, et al (2005) High frequency of human enterovirus species C circulation in Madagascar. J Clin Microbiol 43: 242–249. 15634978 10.1128/JCM.43.1.242-249.2005 PMC540130

[8] Sadeuh-Mba SA, Bessaud M, Massenet D, Joffret ML, Endegue MC, et al (2013) High frequency and diversity of species C enteroviruses in cameroon and neighboring countries. J Clin Mirobiol 51: 759–770. 10.1128/JCM.02119-12 PMC3592076 23254123

[9] Silva PA, Stark K, Mockenhaupt FP, Reither K, Weitzel T, et al (2008) Molecular characterization of enteric viral agents from children in northern region of Ghana. J Med Virol 80: 1790–1798. 18712819 10.1002/jmv.21231

[10] Khetsuriani N, Lamonte-Fowlkes A, Oberst S, Pallansch MA (2006) Enterovirus surveillance–United States, 1970–2005. MMWR Surveill Summ 55: 1–20. 16971890

[11] Tan C, Ninove L, Gaudart J, Nougairede A, Zandotti C, et al (2011) A retrospective overview of enterovirus infection diagnosis and molecular epidemiology in the public hospitals of Marseille, France (1985–2005). PloS one 6: e18022. 21437207 10.1371/journal.pone.0018022 PMC3060927

[12] Antona D, Leveque N, Chomel J, Dubrou S, Levy-Bruhl D, et al (2007) Surveillance of enteroviruses in France, 2000–2004. Eur J Clin Microbiol Infect Dis 26: 403–412. 17534678 10.1007/s10096-007-0306-4

[13] Daleno C, Piralla A, Scala A, Baldanti F, Greenberg D, et al (2013) Full Genome Sequence of a Novel Human Enterovirus C (EV-C118) Isolated from Two Children with Acute Otitis Media and Community-Acquired Pneumonia in Israel. Genome Announc 1: 00121–00112. 10.1128/genomeA.00121-12 PMC3569292 23405305

[14] Lukashev AN, Drexler JF, Kotova VO, Amjaga EN, Reznik VI, et al (2012) Novel serotypes 105 and 116 are members of distinct subgroups of Human enterovirus C. J Gen Virol 93: 2357–2362. 22894922 10.1099/vir.0.043216-0

[15] Pankovics P, Boros A, Szabo H, Szekely G, Gyurkovits K, et al (2012) Human enterovirus 109 (EV109) in acute paediatric respiratory disease in Hungary. Acta Microbiol Immunol Hung 59: 285–290. 22750788 10.1556/AMicr.59.2012.2.13

[16] Smura T, Blomqvist S, Hovi T, Roivainen M (2009) The complete genome sequences for a novel enterovirus type, enterovirus 96, reflect multiple recombinations. Arch Virol 154: 1157–1161. 19526351 10.1007/s00705-009-0418-5

[17] Tapparel C, Junier T, Gerlach D, Van-Belle S, Turin L, et al (2009) New respiratory enterovirus and recombinant rhinoviruses among circulating picornaviruses. Emerg Infect Dis 15: 719–726. 19402957 10.3201/eid1505.081286 PMC2687021

[18] Bessaud M, Pillet S, Ibrahim W, Joffret ML, Pozzetto B, et al (2012) Molecular characterization of human enteroviruses in the central african republic: uncovering wide diversity and identification of a new human enterovirus a71 genogroup. J Clin microbiol 50: 1650–1658. 22337981 10.1128/JCM.06657-11 PMC3347117

[19] Bingjun T, Yoshida H, Yan W, Lin L, Tsuji T, et al (2008) Molecular typing and epidemiology of non-polio enteroviruses isolated from Yunnan Province, the People's Republic of China. J Med Virol 80: 670–679. 18297723 10.1002/jmv.21122

[20] Harvala H, Van Nguyen D, McIntyre C, Ahuka-Mundeke S, Mpoudi Ngole E, et al (2013) Co-circulation of enteroviruses between apes and humans. J Gen Virol 4: 059048–059040. 10.1099/vir.0.059048-0 PMC4093782 24189620

[21] Junttila N, Leveque N, Kabue JP, Cartet G, Mushiya F, et al (2007) New enteroviruses, EV-93 and EV-94, associated with acute flaccid paralysis in the Democratic Republic of the Congo. J Med Virol 79: 393–400. 17311342 10.1002/jmv.20825

[22] Fuentes-Marins R, Rodriguez AR, Kalter SS, Hellman A, Crandell RA (1963) Isolation of enteroviruses from the “normal” baboon (papio doguera). J Bacteriol 85: 1045–1050. 14043993 10.1128/jb.85.5.1045-1050.1963 PMC278282

[23] Heberling RL, Cheever FS (1965) Characteristics of the growth cycles of four simian enteroviruses (SV2, SV6, SV42, SV49). Proc Soc Exp Biol Med 120: 825–828. 4285648 10.3181/00379727-120-30666

[24] Hoffert WB, Bates ME, Cheever FS (1958) Study of enteric viruses of simian origin. Am J Hyg 68: 15–30. 13559208 10.1093/oxfordjournals.aje.a119946

[25] Hull RN, Minner JR, Smith JW (1956) New viral agents recovered from tissue cultures of monkey kidney cells. I. Origin and properties of cytopathogenic agents S.V.1, S.V.2, S.V.4, S.V.5, S.V.6, S.V.11, S.V.12 and S.V.15. Am J Hyg 63: 204–215. 13302209 10.1093/oxfordjournals.aje.a119804

[26] Kalter SS (1982) Enteric Viruses of Nonhuman Primates. Vet Pathol 19: 33–43. 6293149

[27] Rodriguez A, Kalter S, Heberling R, Helmke R, Guajardo J (1977) Viral infections of the captive Kenya baboon (Papio cynocephalus): a five-year epidemiologic study of an outdoor colony. Lab Anim Sci 27: 356–371. 195135

[28] Kalter SS, Kim CS, Sueltenfuss EA (1967) Further characterization of agents isolated from normal baboon (Papio sp.). J Infect Dis 117: 301–306. 4965335 10.1093/infdis/117.4.301

[29] Malherbe H, Harwin R (1963) The cytopathic effects of vervet monkey viruses. S Afr Med J 37: 407–411. 13932505

[30] Oberste MS, Maher K, Pallansch MA (2002) Molecular Phylogeny and Proposed Classification of the Simian Picornaviruses. J Virol 76: 1244–1251. 11773400 10.1128/JVI.76.3.1244-1251.2002 PMC135860

[31] Oberste MS, Maher K, Pallansch MA (2003) Genomic evidence that simian virus 2 and six other simian picornaviruses represent a new genus in Picornaviridae. Virology 314: 283–293. 14517081 10.1016/s0042-6822(03)00420-3

[32] Knowles NJ, Hovi T, Hyypiä T, King AMQ, Lindberg AM, et al.. (2012) Picornaviridae. In Virus Taxonomy: Classification and Nomenclature of Viruses: Ninth Report of the International Committee on Taxonomy of Viruses. London: Academic Press. pp 855–880.

[33] Oberste MS, Maher K, Pallansch MA (2007) Complete genome sequences for nine simian enteroviruses. J Gen Virol 88: 3360–3372. 18024906 10.1099/vir.0.83124-0

[34] Harvala H, Sharp CP, Ngole EM, Delaporte E, Peeters M, et al (2011) Detection and genetic characterization of enteroviruses circulating among wild populations of chimpanzees in Cameroon: relationship with human and simian enteroviruses. J Virol 85: 4480–4486. 21345956 10.1128/JVI.02285-10 PMC3126250

[35] Neel C, Etienne L, Li Y, Takehisa J, Rudicell RS, et al (2010) Molecular epidemiology of simian immunodeficiency virus infection in wild-living gorillas. J Virol 84: 1464–1476. 19906908 10.1128/JVI.02129-09 PMC2812320

[36] Van Heuverswyn F, Li Y, Neel C, Bailes E, Keele B, et al (2006) Human immunodeficiency viruses: SIV infection in wild gorillas. Nature 444: 164. 17093443 10.1038/444164a

[37] Bolanaki E, Kottaridi C, Dedepsidis E, Kyriakopoulou Z, Pliaka V, et al (2008) Direct extraction and molecular characterization of enteroviruses genomes from human faecal samples. Mol Cell Probes 22: 156–161. 18378420 10.1016/j.mcp.2007.12.001

[38] Nix WA, Jiang B, Maher K, Strobert E, Oberste MS (2008) Identification of enteroviruses in naturally infected captive primates. J Clin Microbiol 46: 2874–2878. 18596147 10.1128/JCM.00074-08 PMC2546737

[39] Nix WA, Oberste MS, Pallansch MA (2006) Sensitive, seminested PCR amplification of VP1 sequences for direct identification of all enterovirus serotypes from original clinical specimens. J Clin Microbiol 44: 2698–2704. 16891480 10.1128/JCM.00542-06 PMC1594621

[40] Guillot S, Caro V, Cuervo N, Korotkova E, Combiescu M, et al (2000) Natural Genetic Exchanges between Vaccine and Wild Poliovirus Strains in Humans. J Virol 74: 8434–8443. 10954543 10.1128/jvi.74.18.8434-8443.2000 PMC116354

[41] Bessaud M, Jegouic S, Joffret ML, Barge C, Balanant J, et al (2008) Characterization of the genome of human enteroviruses: design of generic primers for amplification and sequencing of different regions of the viral genome. J Virol Methods 149: 277–284. 18329732 10.1016/j.jviromet.2008.01.027

[42] Oberste MS (2004) Complete genome sequences of all members of the species Human enterovirus A. J Gen Virol 85: 1597–1607. 15166444 10.1099/vir.0.79789-0

[43] Keele BF, Van Heuverswyn F, Li Y, Bailes E, Takehisa J, et al (2006) Chimpanzee reservoirs of pandemic and nonpandemic HIV-1. Science 313: 523–526. 16728595 10.1126/science.1126531 PMC2442710

[44] van der Kuyl A, Kuiken C, Dekker J, Goudsmit J (1995) Phylogeny of African monkeys based upon mitochondrial 12S rRNA sequences. J Mol Evol 40: 173–180. 7535363 10.1007/BF00167111

[45] Hiroshige K, Soejima M, Nishioka T, Kamimura S, Koda Y (2009) Simple and sensitive method for identification of human DNA by allele-specific polymerase chain reaction of FOXP2. J Forensic Sci 54: 857–861. 19457146 10.1111/j.1556-4029.2009.01063.x

[46] Brown BA, Maher K, Flemister MR, Naraghi-Arani P, Uddin M, et al (2009) Resolving ambiguities in genetic typing of human enterovirus species C clinical isolates and identification of enterovirus 96, 99 and 102. J Gen Virol 90: 1713–1723. 19264596 10.1099/vir.0.008540-0

[47] Tamura K, Peterson D, Peterson N, Stecher G, Nei M, et al (2011) MEGA5: molecular evolutionary genetics analysis using maximum likelihood, evolutionary distance, and maximum parsimony methods. Mol Biol Evol 28: 2731–2739. 21546353 10.1093/molbev/msr121 PMC3203626

[48] Guindon S, Dufayard JF, Lefort V, Anisimova M, Hordijk W, et al (2010) New algorithms and methods to estimate maximum-likelihood phylogenies: assessing the performance of PhyML 3.0. Syst Biol 59: 307–321. 20525638 10.1093/sysbio/syq010

[49] Kimura M (1980) A simple method for estimating evolutionary rates of base substitutions through comparative studies of nucleotide sequences. J Mol Evol 16: 111–120. 7463489 10.1007/BF01731581

[50] Hasegawa M, Kishino H, Yano T (1985) Dating of the human-ape splitting by a molecular clock of mitochondrial DNA. J Mol Evol 22: 160–174. 3934395 10.1007/BF02101694

[51] Lee MS, Chiang PS, Luo ST, Huang ML, Liou GY, et al (2012) Incidence rates of enterovirus 71 infections in young children during a nationwide epidemic in Taiwan, 2008–09. PLoS Negl Trop Dis 6: e1476. 22348156 10.1371/journal.pntd.0001476 PMC3279337

[52] Tan X, Huang X, Zhu S, Chen H, Yu Q, et al (2011) The persistent circulation of enterovirus 71 in People's Republic of China: causing emerging nationwide epidemics since 2008. PloS one 6: e25662. 21980521 10.1371/journal.pone.0025662 PMC3181342

[53] Bessaud M, Razafindratsimandresy R, Nougairede A, Joffret ML, Deshpande JM, et al (2014) Molecular comparison and evolutionary analyses of VP1 nucleotide sequences of new African human enterovirus 71 isolates reveal a wide genetic diversity. PloS one 9: e90624. 24598878 10.1371/journal.pone.0090624 PMC3944068

[54] Bessaud M, Joffret ML, Holmblat B, Razafindratsimandresy R, Delpeyroux F (2011) Genetic relationship between cocirculating Human enteroviruses species C. PloS one 6: e24823. 21931857 10.1371/journal.pone.0024823 PMC3171481

[55] Oberste MS, Feeroz MM, Maher K, Nix WA, Engel GA, et al (2012) Characterizing the picornavirus landscape among synanthropic nonhuman primates in Bangladesh, 2007–2008. J Virol 24: 24. 10.1128/JVI.00837-12 PMC3536389 23097448

[56] Oberste MS, Feeroz MM, Maher K, Nix WA, Engel GA, et al (2012) Naturally acquired picornavirus infections in nonhuman primates at the Dhaka Zoo. J Virol 24: 24. 10.1128/JVI.00838-12 PMC3536375 23097447

[57] Oberste MS, Maher K, Michele SM, Belliot G, Uddin M, et al (2005) Enteroviruses 76, 89, 90 and 91 represent a novel group within the species Human enterovirus A. J Gen Virol 86: 445–451. 15659764 10.1099/vir.0.80475-0

[58] Harvala H, McIntyre C, Imai N, Clasper L, Djoko C, et al (2012) High seroprevalence of enterovirus infections in apes and old world monkeys. Emerg Infect Dis 18: 283–286. 22305156 10.3201/eid1802.111363 PMC3310466

[59] Smura T, Blomqvist S, Paananen A, Vuorinen T, Sobotova Z, et al (2007) Enterovirus surveillance reveals proposed new serotypes and provides new insight into enterovirus 5′-untranslated region evolution. J Gen Virol 88: 2520–2526. 17698662 10.1099/vir.0.82866-0

