# Supplementary figures and images for: Characterization of Enteroviruses from Non-Human Primates in Cameroon Revealed Virus Types Widespread in Humans along with Candidate New Types and Species
Source: PLoS Negl Trop Dis. 2014 Jul 31;8(7):e3052. doi: 10.1371/journal.pntd.0003052 (PMC4117447; doi:10.1371/journal.pntd.0003052)

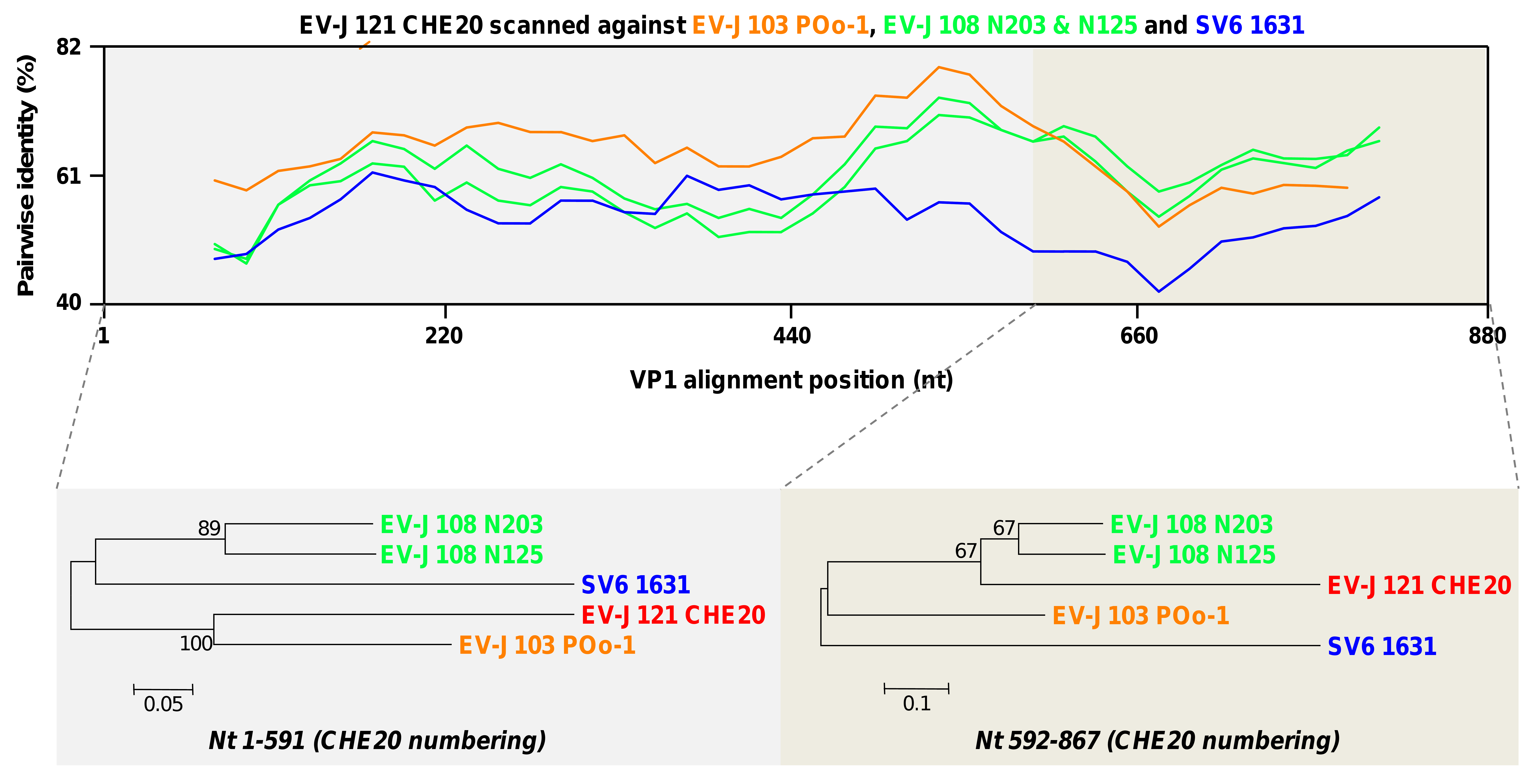

Supplement: Figure S1 — Comparative analysis of the full-length VP1 sequences of SV6, EV-J103, EV-J108, and the newly sequenced EV-J121 types. The similarity plots were generated using SimPlot version 3.5.1 with 100 nucleotide (nt) windows, 20 nt increments, and the Kimura 2-parameter method with a transition-transversion ratio of 8.0. Partial VP1 based trees depicting the relationships between the strains considered are presented at the bottom of the corresponding target portion. (TIF) [file pntd.0003052.s001.tif]
