# Supplementary material for: Characterization of Enteroviruses from Non-Human Primates in Cameroon Revealed Virus Types Widespread in Humans along with Candidate New Types and Species
Source: PLoS Negl Trop Dis. 2014 Jul 31;8(7):e3052. doi: 10.1371/journal.pntd.0003052 (PMC4117447; doi:10.1371/journal.pntd.0003052)
Supplement: Table S1 — Summary of the detection and sequencing in the 5′UTR, VP1 and 3Dpol regions of enteroviruses among wild and captive non-human primates. (DOCX) [file pntd.0003052.s002.docx]

**Table S1. Summary of the detection and sequencing in the 5’UTR, VP1 and 3D^pol^ regions of enteroviruses among wild and captive non-human primates.**

|  |  | **Primates** | | |  | **GenBank accession numbers**^c^ | | | | |
| --- | --- | --- | --- | --- | --- | --- | --- | --- | --- | --- |
| **viruses**^a^ |  | **Species**^b^ |  | **living  setting** |  | **5'UTR** |  | **VP1**^d^ |  | **3D^pol^** |
| **CHI-1A** | | chimpanzee |  | wild |  | KF614478 |  | n/a |  | n/a |
| **CHI-1B** | | chimpanzee |  | wild |  | KF614479 |  | n/a |  | n/a |
| **CHI-2A** | | *Pan t. troglodytes* |  | wild |  | KF614480 |  | n/a |  | n/a |
| CHB2 | | *Pan t. troglodytes* |  | wild |  | KF614472 |  | **KF541633** |  | KF648600 |
| CHB5 | | *Pan t. troglodytes* |  | wild |  | KF614473 |  | **KF541631** |  | n/a |
| CHB6 | | *Pan t. troglodytes* |  | wild |  | KF614474 |  | KF541649 |  | n/a |
| CHB7 | | *Pan t. troglodytes* |  | wild |  | KF614475 |  | KF541648 |  | KF648601 |
| CHB8 | | *Pan t. troglodytes* |  | wild |  | KF614476 |  | **KF541632** |  | n/a |
| CHE20 | | *Pan t. troglodytes* |  | wild |  | KF614477 |  | KF541639 |  | KF648606 |
| **CHJ02** | | *Pan t. troglodytes* |  | wild |  | n/a |  | n/a |  | KF648607 |
| GORJ01 |  | *Gorilla gorilla* |  | wild |  | n/a |  | **KF541630** |  | n/a |
| MDRH7 | | *Mandrillus sphinx* |  | wild |  | KF614488 |  | n/a |  | n/a |
| RCMH11 | | *Mandrillus sphinx* |  | wild |  | KF614489 |  | n/a |  | KF648616 |
| RCMH4 | | *Mandrillus sphinx* |  | wild |  | KF614491 |  | n/a |  | KF648618 |
| RCMH7 | | *Mandrillus sphinx* |  | wild |  | KF614494 |  | KF541635 |  | KF648602 |
| RCMH3 | | red-caped mangabey |  | wild |  | KF614490 |  | KF541637 |  | KF648617 |
| RCMH5 | | red-caped mangabey |  | wild |  | KF614492 |  | KF541636 |  | KF648619 |
| RCMH6 | | red-caped mangabey |  | wild |  | KF614493 |  | KF541638 |  | KF648620 |
| Z004 | | chimpanzee |  | captive |  | KF614481 |  | KF541646 |  | KF648610 |
| Z036 | | *Pan t. troglodytes* |  | captive |  | KF614482 |  | KF541645 |  | KF648611 |
| Z055 | | *Pan t. troglodytes* |  | captive |  | KF614483 |  | KF541644 |  | KF648612 |
| Z108-1 | | *Pan t. troglodytes* |  | captive |  | KF614487 |  | KF541647 |  | KF648608 |
| Z108-2 |  | *Pan t. troglodytes* |  | captive |  | n/a |  | n/a |  | KF648605 |
| Z033 | | *Pan t. troglodytes* |  | captive |  | KF614495 |  | **KF541627** |  | n/a |
| Z034 | | *Pan t. troglodytes* |  | captive |  | KF614496 |  | **KF541628** |  | KF648621 |
| Z035 | | *Pan t. troglodytes* |  | captive |  | KF614497 |  | **KF541629** |  | KF648622 |
| Z046 | | *Pan t. troglodytes* |  | captive |  | KF614501 |  | KF541634 |  | KF648603 |
| Z110 | | *Pan t. troglodytes* |  | captive |  | KF614507 |  | **KF541626** |  | n/a |
| Z052 | | *Gorilla gorilla* |  | captive |  | KF614502 |  | KF541640 |  | KF648609 |
| Z097 | | *Gorilla gorilla* |  | captive |  | KF614486 |  | KF541641 |  | KF648615 |
| Z098-1 |  | *Gorilla gorilla* |  | captive |  | n/a |  | **KF541625** |  | n/a |
| Z098-2 |  | *Gorilla gorilla* |  | captive |  | KF614504 |  | KF700265 |  | KF648604 |
| Z099 |  | *Gorilla gorilla* |  | captive |  | KF614505 |  | KF700264 |  | n/a |
| **Z043** |  | *C. neglectus* |  | captive |  | KF614498 |  | n/a |  | n/a |
| Z045 |  | *C. neglectus* |  | captive |  | KF614500 |  | n/a |  | n/a |
| Z044 |  | *Chlorocebus tantalus* |  | captive |  | KF614499 |  | n/a |  | n/a |
| Z057 |  | *Mandrillus sphinx* |  | captive |  | KF614484 |  | KF541643 |  | KF648613 |
| Z088 |  | *Mandrillus sphinx* |  | captive |  | KF614485 |  | KF541642 |  | KF648614 |
| Z083 | | *Papios anubis* |  | captive |  | KF614503 |  | n/a |  | n/a |
| Z109 | | *Papios anubis* |  | captive |  | KF614506 |  | n/a |  | n/a |

^a^, Viruses Z108-1 & Z108-2 in one hand and Z098-1 & Z098-2 in the other hand were detected in the same chimpanzee (Z108) and gorilla (Z098) sample, respectively. Viruses that showed divergent 5’UTR and 3D^pol^ sequences are indicated in bold.

^b^, species designated with scientific names were confirmed by the analysis of a portion of simian mitochondrial DNA sequences; Pan t. troglodytes, *Pan troglodytes troglodytes*; C. neglectus, *Cercopithecus neglectus*.

^c^, n/a, not applicable because of low amplification signals (VP1 region) or negative PCR results (5’UTR and 3D^pol^ regions).

^d^, Accession numbers for partial VP1 sequences are highlighted in bold.
